# Amphidinol 22, a New Cytotoxic and Antifungal Amphidinol from the Dinoflagellate *Amphidinium carterae*

**DOI:** 10.3390/md17070385

**Published:** 2019-06-27

**Authors:** Kevin A. Martínez, Chiara Lauritano, Dana Druka, Giovanna Romano, Teresa Grohmann, Marcel Jaspars, Jesús Martín, Caridad Díaz, Bastien Cautain, Mercedes de la Cruz, Adrianna Ianora, Fernando Reyes

**Affiliations:** 1 Department of Marine Biotechnology, Stazione Zoologica Anton Dohrn, 80121 Naples, Italy; 2 Marine Biodiscovery Centre, Department of Chemistry, University of Aberdeen, Aberdeen AB24 3UE, Scotland, UK; 3 The Rowett Institute, University of Aberdeen, Aberdeen AB25 2ZD, Scotland, UK; 4 Fundación MEDINA, Centro de Excelencia en Investigación de Medicamentos Innovadores en Andalucía, Avda. del Conocimiento 34, 18016 Granada, Spain

**Keywords:** marine microalgae, dinoflagellates, marine natural products (MNPs), bioactive compounds, blue biotechnology, amphidinol, antifungal, anticancer

## Abstract

Due to the unique biodiversity and the physical-chemical properties of their environment, marine microorganisms have evolved defense and signaling compounds that often have no equivalent in terrestrial habitats. The aim of this study was to screen extracts of the dinoflagellate *Amphidinium carterae* for possible bioactivities (i.e., anticancer, anti-inflammatory, anti-diabetes, antibacterial and antifungal properties) and identify bioactive compounds. Anticancer activity was evaluated on human lung adenocarcinoma (A549), human skin melanoma (A2058), human hepatocellular carcinoma (HepG2), human breast adenocarcinoma (MCF7) and human pancreas carcinoma (MiaPaca-2) cell lines. Antimicrobial activities were evaluated against Gram-positive bacteria (*Staphylococcus aureus* MRSA and MSSA), Gram-negative bacteria (i.e., *Escherichia coli* and *Klebsiella pneumoniae*), *Mycobacterium tuberculosis* and the fungus *Aspergillus fumigatus*. The results indicated moderate biological activities against all the cancer cells lines and microorganisms tested. Bioassay-guided fractionation assisted by HRMS analysis allowed the detection of one new and two known amphidinols that are potentially responsible for the antifungal and cytotoxic activities observed. Further isolation, purification and structural elucidation led to a new amphidinol, named amphidinol 22. The planar structure of the new compound was determined by analysis of its HRMS and 1D and 2D NMR spectra. Its biological activity was evaluated, and it displayed both anticancer and antifungal activities.

## 1. Introduction

Many microalgae, including dinoflagellates, are known to produce compounds with a wide range of biological and biochemical properties [1]. The biodiversity of marine phytoplankton species leads to a great metabolic variety that renders them a huge reservoir of new bioactive compounds with multiple possible pharmaceutical applications [2] (e.g. cytotoxic, anticancer, antibiotic, antifungal, immunosuppressant and neurotoxic activities [3,4,5,6,7,8,9,10]). Bioactive compounds of microalgal origin can be sourced directly from primary metabolism (e.g. proteins, fatty acids, vitamins and pigments) or can be synthesized from secondary metabolism. Microalgae are in fact excellent sources/producers of carotenoids, polysaccharides, vitamins, lipids as well as potent neurotoxins [11]. In the last decade, an increasing number of studies have focused their attention on microalgal compounds for the treatment of various human pathologies or for nutraceutical applications [12,13].

*Amphidinium carterae* is an athecate dinoflagellate, found in both temperate and tropical waters [14]. Pagliara and Caroppo [15] showed that when embryos of the sea urchin *Paracentrotus lividus* were reared on a Mediterranean strain of *A. carterae*, there was a 60% embryo growth inhibition after exposure to 3.75 mg/mL of *A. carterae* cell lysate and 100% inhibition with 7.5 mg/mL. They also observed an LC_50_ value of 3.67 mg/mL after 24 h exposure of the brine shrimp *Artemia salina* to the *A. carterae* cell lysate. Shah and co-workers found antioxidant activity at 1 mg/mL of methanolic extract of *A. carterae*, 80% nitric oxide (NO) production inhibition at 50 µg/mL in LPS-induced RAW 264.7 macrophages and 20% reduction in human promyelocytic leukemia (HL-60) cell viability at 50 µg/mL [16]. In addition, it was also reported that an extract from another *Amphidinium* strain had antifungal properties and was able to inhibit the growth of the fungus *Candida albicans* at 32 µg/mL [17].

Until now, various *Amphidinium* strains have displayed different biological activities and chemical diversity [17,18,19,20,21,22,23]. Various compounds have been isolated from these strains, including the amphidinols (AM), amphirionins, karatungiols and more than 45 cytotoxic macrolides, known as amphidinolides. Most of the mentioned compounds present polyketidic skeletons, but it seems that not all of the *Amphidinium* strains possess the enzymatic machinery responsible for polyketide synthesis [24,25].

The aim of this study was to screen *A. carterae* (clone FE102) for various bioactivities useful for the treatment of human pathologies (i.e., anticancer, antibacterial and antifungal properties) and isolate potential active metabolites. In the transcriptome of the same clone, we recently found the sequence coding β-ketosynthase [26], an enzyme involved in polyketide synthesis, suggesting the production of potential active secondary metabolites. Raw extracts from *A. carterae* were tested against various bioactivity platforms including both human and bacterial cells. We evaluated the anti-bacterial activity on Gram-positive bacteria (*Staphylococcus aureus* MRSA, *Staphylococcus aureus* MSSA), Gram-negative bacteria (*Escherichia coli* and *Klebsiella pneumoniae*) and *Mycobacterium tuberculosis*. The antifungal activity was assessed on *Aspergillus fumigatus*. Anticancer activity was also evaluated against a panel of five different cancer cell lines (i.e., human lung carcinoma A549 ATCC^®^ CCL-185™, human skin melanoma A2058 ATCC^®^ CRL-11147™, hepatocyte carcinoma HepG2 ATCC^®^ HB-8065™, breast adenocarcinoma MCF7 ATCC^®^ HTB-22™ and pancreas carcinoma MiaPaca-2 ATCC^®^ CRL-1420™). Bioassay-guided fractionation followed by HRMS analyses yielded the new amphidinol 22. The amphidinols are a family of bioactive polyketides with a well-known antifungal and hemolytic activity, as reported in the literature [17,21,27,28]. Espiritu et al. have also reported that amphidinol 2 (AM2) displayed anticancer properties [29]. Amphidinol 22 biological activity was evaluated and it showed both anticancer and antifungal activity.

## 2. Experimental Section

### 2.1. Cell Culturing and Harvesting

*A. carterae* (CCMP1314) was grown in Keller medium [30] in ten-liter polycarbonate carboys (each experiment was performed in triplicate) constantly bubbled with air filtered through 0.2 µm membrane filters. Cultures were kept in a climate chamber at 20 °C on a 12:12 h light:dark cycle at 110 µmol photons m^−2^ s^−1^. Initial cell concentrations were about 5000 cells/mL for each experiment and culture growth rate was monitored, using the equation for net growth estimates [31]. For the isolation of the pure compound, fifteen replicates of ten-liter cultures have been used. The biomass was harvested during the stationary phase (in the same day and at the same time of the day for each replicate to avoid possible interference by intrinsic circadian rhythms) by centrifugation for 10 min at 4 °C at 2300 rpm (Beckman Coulter Allegra^®^ 6R centrifuge). Microalgal biomasses were kept at −80 °C until chemical extraction. 

### 2.2. Chemical Extraction 

Methanol was filled into the tubes to reach two times the volume of the biomass. The tubes were vortexed to ensure that methanol completely soaked the material that was then placed into a Kuhner ISF4-X Climo-Shaker for 3 h at 20 °C. The tubes were then centrifuged at 3000 rpm for 20 min, and the supernatant was transferred to 40 mL EPA vials (dispolab) and evaporated under nitrogen stream. Aliquots from the dried extracts were lyophilized and dissolved in DMSO for testing and HPLC-UV-MS analysis. The extract yield of the *A. carterae* broth was 50 mg/L approximately.

### 2.3. Anticancer Assays

Colorimetric MTT ((3-(4,5-Dimethylthiazol-2-yl)-2,5-diphenyltetrazolium bromide)) assays were carried out to assess the cell viability of the samples against a panel of five different cancer cell lines (i.e., human lung carcinoma A549 ATCC^®^ CCL-185™, human skin melanoma A2058 ATCC^®^ CRL-11147™, hepatocyte carcinoma HepG2 ATCC^®^ HB-8065™, breast adenocarcinoma MCF7 ATCC^®^ HTB-22™ and pancreas carcinoma MiaPaca-2 ATCC^®^ CRL-1420™). All cells were obtained from the American Type Culture Collection (ATCC, Manassas, VA, USA). A549 cells were grown in Ham′s F12K medium with 2 mM Glutamine, 10% Fetal Bovine Serum (FBS), 100 U/mL penicillin and 100 µg/mL streptomycin. A2058 and HepG2 were grown in ATCC formulated Eagle’s M essential medium (MEM) with 10% FBS, 2 mM l-glutamine, 1 mM sodium pyruvate and 100 µM MEM non-essential amino acids. MCF-7 cells were grown in the previous medium supplemented with 0.01 mg/mL of bovine insulin. MiaPaca-2 cells were grown in Dulbecco’s Modified Eagle’s Medium (DMEM) with 10% FBS, 100U/mL penicillin and 100 µg/mL streptomycin. The bioassays were performed as reported by Audoin et al. [32]. The anticancer activity was assessed after 72 h of treatment with amphidinol 22 at the concentrations 0.098, 0.195, 0.391, 0.781, 1.563, 3.125, 6.250, 12.5, 25 and 50 µM. The raw algal extract was tested at 175 µg/mL.

### 2.4. Antifungal Assays

The fungi *C. albicans* ATCC64124 and *A. fumigatus* ATCC46645 were used as test organisms to assess antifungal activity as reported in Audoin et al. [32]. For *C. albicans* the growth inhibition was calculated measuring the OD at 612 nm, while for *A. fumigatus* it was based on the fluorescence development derived from the conversion of resazurin to resorufin (excitation 570 nm and emission 600 nm). The antifungal activity was assessed after 20–30 h of treatment with amphidinol 22 at the concentrations 0.075, 0.15, 0.3, 0.6, 1.25, 2.5, 5, 10, 20 and 40 µM. The raw algal extract was tested at 560 µg/mL.

### 2.5. Antibacterial Assays

A panel of five different bacteria (i.e., the Gram-negative bacteria *E. coli* ATCC25922 and *K. pneumoniae* ATCC700603, and Gram-positive bacteria *S. aureus* MRSA MB5393 and MSSA ATCC29213) were used as test organisms for the antibacterial MIC (minimum inhibitory concentration) assays that were performed as reported in Audoin et al. [32]. The antibacterial activity was assessed after 20 h of treatment with amphidinol 22 at the concentrations 0.075, 0.15, 0.3, 0.6, 1.25, 2.5, 5, 10, 20 and 40 µM. The raw algal extract was tested at 560 µg/mL. The antitubercular activity of the samples against *M. tuberculosis* H37Ra was determined using the REMA method [33].

### 2.6. General Chemical Analysis Procedures

Samples were analyzed by HPLC-UV-HRMS on an Agilent 1200 RR coupled to a Bruker maXis time-of-flight spectrometer with electrospray ionization, as reported by Martin et al. [34]. Mass spectra were collected as full scans from 50 *m*/*z* to 2000 *m*/*z*. Data were analyzed using the platform available at Fundación MEDINA [35] and compared with the data available in the Dictionary of Marine Natural Products database [36] and PubChem [37]. NMR spectra were acquired using a Bruker Avance 500 MHz spectrometer with a pulsed field gradient and referenced to signal solvent signals (CD3OD, at δH 3.31 and δC 49.0 ppm). The solvents used were HPLC grade. 

### 2.7. Fractionation

The crude microalgal extract (5.67 g) was fractionated through reverse phase C18 (110 mm × 40 mm column) flash chromatography to separate the extract into 80 different fractions using a gradient of H2O (Solvent A) and CH3CN (Solvent B). The method went from 5% B to 100% B in 40 min and it was maintained at 100% B for another 40 min. The flow rate was 18 mL/min and the wavelengths selected were 210 and 260 nm, according to the data from HPLC-UV-MS. The fractions 19 and 20 (17.73 mg, eluted at 48% B) were pooled and further fractionated by semi-preparative reversed-phase HPLC-DAD (column Zorbax SB-C8, 4.6 mm × 150 mm, 5 µm particle size) with a flow of 3.6 mL/min and a H2O:CH3CN gradient (75:25 to 68:32 in 36 min) as eluent. Amphidinol 18 (identified by HPLC-UV-MS) and amphidinol 22 were isolated. Amphidinol 22 was obtained as a pale yellow solid (1.4 mg) and it was tested for anticancer, antifungal and antibacterial activity using 10-point serial dilutions (1:1 dilutions), with initial concentrations of 50 µM in the case of the anticancer assays, and 40 µM in the case of the antibacterial and antifungal assays.

### 2.8. Amphidinol 22

Pale yellow, amorphous solid; UV (MeOH) *λ*_max_ 282, 270 and 260 nm; NMR data available (CD3OD), see Table 1; HRESIMS *m*/*z* 1662.9705 [M + NH4]^+^ (calcd for C84H144O31N^+^, 1662.9717, Δ −0.7 ppm); 1645.9515 [M + H]^+^ (calcd for C84H141O31^+^, 1645.9451, Δ 3.9 ppm); 1627.9371 [M−H2O + H]^+^ (calcd for C84H139O30^+^, 1627.9346, Δ 1.5 ppm). 1H NMR (500 MHz), ^13^C NMR (125 MHz), HSQC, COSY, HMBC, NOESY, LC-UV trace and UV and HRESIMS spectra of amphidinol 22, expansions of the HRESIMS spectrum, and the tabulated 2D NMR data are available in the supplementary information (Figures S1–S8 and Table S9, respectively).

### 2.9. Statistical Analysis

Statistical differences between treated and control cells for all the assays performed in this study were determined by Student’s *t*-test using GraphPad Prim statistic software, V4.00 (GraphPad Software, San Diego, California, USA). Data were considered significant when at least *p* was <0.05 (* for *p* < 0.05, ** for *p* < 0.01, and *** for *p* < 0.001).

## 3. Results and Discussion

### 3.1. Preliminary Bioactivity Screening of Raw Extract and HPLC-UV-MS Analysis

The isolation of new bioactive metabolites was performed following a bioassay-guided fractionation approach supported by HRMS analysis. After extraction of the *A. carterae* biomass, the crude extract was tested against a panel of different cancer cell lines, bacteria and fungi. For each assay, three biological replicates were tested and two technical replicates per sample were performed. Table 2 summarizes the outputs from the bioassays.

The crude methanol extract from *A. carterae* seemed to possess unspecific bioactivity and a clear toxicity profile, considering all the results from the cells and microorganisms tested. This was not surprising, since marine dinoflagellates belonging to the genus *Amphidinium* are well-known producers of different toxins [38], such as the amphidinolides [18].

Early HPLC-UV-MS analysis on the crude extracts revealed the presence of three possible amphidinols that were singled out because of their characteristic UV pattern (showing maxima of absorbance at 260, 270 and 280 nm due to a conjugated triene substructure), retention time and its *m/z* values, which were close to some of the already known amphidinols [17,21]. The presence of these compounds may explain the antifungal and anticancer activities observed within the crude extract.

**Figure 1:**
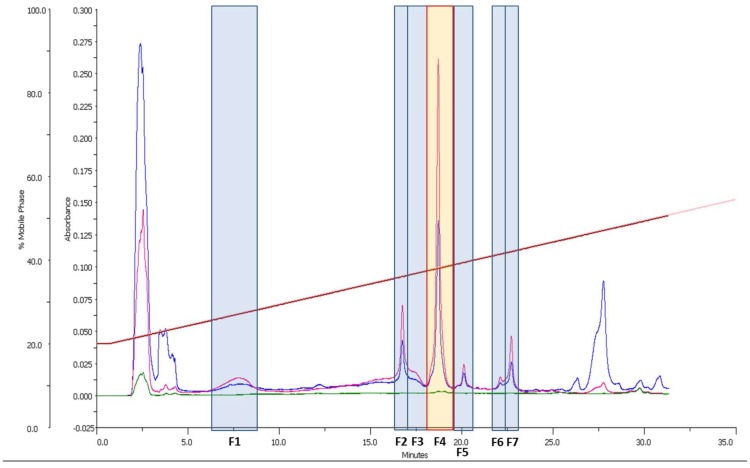
Fractions from the *A. carterae* extract aliquot. The fraction marked in yellow (F4) was active against *A. fumigatus* and *C. albicans.* Wavelengths selected were 210 nm (in blue), 260 nm (in pink) and 310 nm (in green). High 260 nm absorbance indicates the presence of the triene substructure in the amphidinols.

A 10 mg aliquot from the crude extract was fractionated by semipreparative HPLC-DAD as reported in the methods section. Seven fractions (F1 to F7) were collected, dried, dissolved in 100% DMSO and tested against *A. fumigatus* and *C. albicans* (Figure 1).

HPLC-UV-MS analysis of the fractions and dereplication of the molecular formulae obtained led to the identification of F1 and F4 as amphidinol 19 (AM19, exact mass 1438.7894) and amphidinol 18 (AM18, exact mass 1358.8326), respectively (Figure 1). These two compounds were already discovered and tested by Nuzzo et al. [17]. In particular, AM18 displayed antifungal activity against *C. albicans* at 9 µg/mL, but AM19 was not active. F2 was identified as an unknown compound related to amphidinols with a relatively high experimental accurate mass of 1644.9437. The largest amphidinols described so far were amphidinol 20 (AM20) and amphidinol 21 (AM21) [21], and their masses did not match with the mass obtained for the compound in F2. Fractions F3, F5, F6 and F7 corresponded to other amphidinol-related minor compounds.

The full *A. carterae* crude extract (5.7 g) was then fractionated through reverse phase C18 flash chromatography. The compound detected in F2 (1.4 mg, accurate mass 1644.9437), now named amphidinol 22, was isolated together with AM18 from the flash fractions 19 and 20 by semipreparative reversed-phase HPLC.

### 3.2. Structure Identification

Amphidinol 22 was isolated as a pale yellow, amorphous solid. The data obtained from HPLC-UV-MS analysis confirmed UV absorption maxima at 260, 270 and 280 nm, corresponding to the presence of a conjugated triene in the structure, typical among the amphidinols. The molecular formula of amphidinol 22 was deduced from the observed ammonium adduct [M + NH_4_]^+^ (*m/z* 1662.9705, calculated *m/z* 1662.9717), indicative of a molecular formula C_84_H_140_O_31_ (degrees of unsaturation = 15).

**Figure 2:**
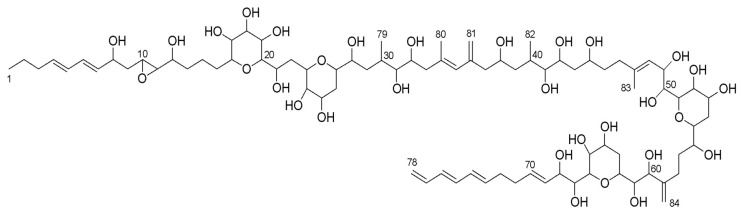
Planar structure of amphidinol 22.

Its ^13^C NMR spectrum revealed the presence of 79 different carbon signals (anticipating the overlapping of some signals) of which 20 signals corresponded to *sp^2^* carbons, covering 10 out of the 15 unsaturations predicted by the molecular formula and suggesting the presence of five cycles. A total of 33 carbon signals corresponded to oxygenated carbons in a region between δ55.11 and δ82.36 ppm. Two of them presented a particularly shielded chemical shift (δ_C_ of 55.11 and 62.35), typical in epoxide groups. The ^1^H NMR spectrum showed a high degree of signal overlap, especially in the region of the oxygenated protons (δ_H_ from 3 to 4 ppm), hence the multiplicity of only a few signals in the outermost regions was easily determined. The previous information together with the 2D-NMR hetero/homo-nuclear experiments performed (HSQC, COSY, HMBC and NOESY), allowed the identification of the planar structure of amphidinol 22 (Figure 2) by linking spin systems and giving information to overcome problems related to overlapped signals.

**Figure 3:**
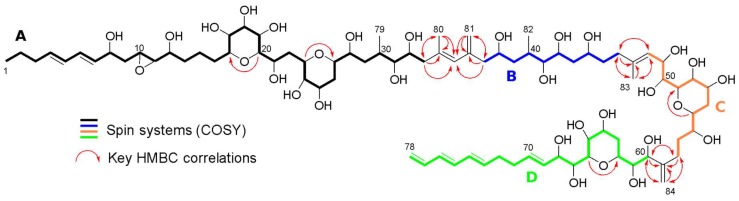
Spin systems and key HMBC correlations of amphidinol 22. The spin systems appear in different colors as follows: **A** (black), **B** (blue), **C** (orange) and **D** (green).

The HSQC spectrum revealed that the carbon signals at δ_C_ 30.41 (2 CH_2_) and 33.59 (CH and CH_2_), and δ 36.68 (2CH_2_), 74.63 (2 CH) and 76.77 (2 CH) each accounted for two carbons, confirming the presence of 84 carbons in the molecule, and also confirmed that δ 136.85, δ 139.30, δ 144.65 and δ 151.35 were signals corresponding to four *sp^2^* quaternary carbons. A comparative approach with the data available from AM18 and AM19 [17], together with the COSY data from our molecule and the HMBC correlations when the overlapping in the ^1^H NMR signals was found (Table S9, Supplementary information), were used to establish the presence of four spin systems in the proposed structure (Figure 3): A (from H1 to H33), B (from H37 to H46), C (from H48 to H58) and, finally, D (from H60 to H78). Almost all COSY signals within the spin systems were well visible and, in addition, long distance COSY correlations were observed between H35, H80 and H81, confirming their proximity. The similarities of the chemical shifts with AM18 and the HMBC correlations confirmed the basic structure.

Key HMBC correlations (Figure 3) allowed to link all the spin systems present in the structure. The cross-peaks at δ 2.29/18.51 (H33/C80), δ 2.29/129.54 (H33/C35), δ 2.29/136.85 (H33/C34), δ 1.84/46.06 (H80/C33), δ 1.84/136.85 (H80/C34) and δ 1.84/129.54 (H80/C35), together with the signals at δ 2.27–2.21/115.77 (H37/C81), δ 2.27–2.21/144.65 (H37/C36), δ 2.27–2.21/129.54 (H37/C35), δ 5.05–4.85/129.54 (H81/C35), δ 5.05–4.85/144.65 (H81/C36) and δ 5.05–4.85/47.72 (H81/C37), allowed to link the spin systems A and B. The HMBC signals H80/C35 and H81/C35, and previously reported COSY signals confirmed the position of CH-35.

Spin system B and C were linked by the CH_2_-46 cross-peaks in the HMBC at δ 2.20–2.11/126.24 (H46/C48), δ 2.20–2.11/139.30 (H46/C47) and δ 2.20–2.11/17.39 (H46/C83), in addition to those of the CH-48 signals at δ 5.48/17.39 (H48/C83) and δ 5.48/36.68 (H48/C46). This link was confirmed with the HMBC correlations of the CH_3_-83 signals at δ 1.74/36.68 (H83/C46), δ 1.74/139.30 (H83/C47) and δ 1.74/126.24 (H83/C48).

Spin systems C and D were linked by the HMBC signals of CH_2_-58 at δ 2.41–2.09/151.35 (H58/C59), δ 2.41–2.09/76.77 (H58/C60) and δ 2.41-2.09/113.21 (H58/C84), together with the CH-60 HMBC signals at δ 4.18/151.35 (H60/C59), δ 4.18/27.89 (H60/C58) and δ 4.18/113.21 (H60/C84). This link was confirmed with the cross-peaks displayed by CH_2_-84 signals at δ 5.07–4.98/27.89 (H84/C58), δ 5.07–4.98/151.35 (H84/C59) and δ 5.07–4.98/76.77 (H84/C60).

Finally, HMBC correlations also allowed to localize four ether bridges along the structure by the through-oxygen cross-peaks at δ 3.14/81.54 (H20/C16), δ 4.22/74.63 (H23/C27), δ 3.48/79.21 (H55/C51) and δ 3.74/70.41 (H66/C62).

**Figure 4:**
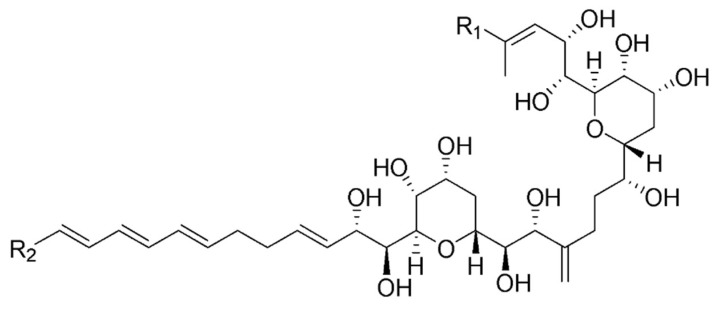
Reported stereochemistry of already known amphidinols in the common substructure with amphidinol 22.

The magnitude of the coupling constants of the signals belonging to the Δ^4^, Δ^6^, Δ^69^, Δ^73^ and Δ^75^ double bonds, higher than 15 Hz in all cases (see Table 1), allowed us to establish their *E* configuration. NOESY signals observed for the pairs H33/H35 and H46/H48 also confirmed the *E* configuration of the Δ^34^ and Δ^47^ double bonds. Although determination of the full the three dimensional structure of the molecule is out of the scope of this report, the configuration of the common substructure in amphidinol 22 might be the same as the one already reported for AM18, AM19, AM20 and AM21 (Figure 4), due to similar chemical shifts and coupling constants around this region [17,21].

### 3.3. Amphidinol 22 Bioactivities

In this section, the anticancer and antimicrobial bioactivities of amphidinol 22 are reported. The compound was evaluated against five different cancer cell lines (i.e., A549, A2058, HepG2, MCF7 and MiaPaca2) and five different microorganisms (i.e., *A. fumigatus*, *C. albicans*, MRSA, MSSA and *M. tuberculosis*).

#### 3.3.1. Anticancer Activity

**Figure 5:**
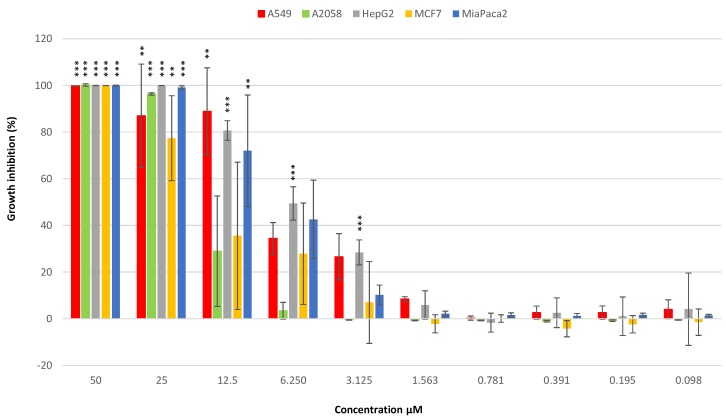
Percentage cell viability inhibition of A549 (lung), A2058 (skin), HepG2 (liver), MCF7 (breast) and Miapaca-2 (pancreas) cancer cell lines after incubation for 72 h with 0.098, 0.195, 0.391, 0.781, 1.563, 3.125, 6.250, 12.5, 25, 50 µM of amphidinol 22 (** for *p* < 0.01 and *** for *p* < 0.001, Student’s *t*-test). Experiments were performed in triplicate.

MTT assays were performed on human lung carcinoma A549 ATCC^®^ CCL-185™, human skin melanoma A2058 ATCC^®^ CRL-11147™, hepatocyte carcinoma HepG2 ATCC^®^ HB-8065™, breast adenocarcinoma MCF7 ATCC^®^ HTB-22™ and pancreas carcinoma MiaPaca-2 ATCC^®^ CRL-1420™ cell lines to assay the potential anticancer activity of amphidinol 22. The compound displayed general cytotoxicity on all the cell lines tested (Figure 5). Its IC_50_ values on A549, A2058, HepG2, MCF7 and MiaPaca-2 cell lines were 8 µM, 16.4 µM, 6.8 µM, 16.8 µM and 8.6 µM, respectively.

The anticancer properties of another member of the amphidinol family, amphidinol 2 (AM2, first isolated by Paul et al. [39]) were already reported by Espiritu et al. [29]. The anticancer activity was evaluated against HCT-116 (colon carcinoma), HT29 (colon adenocarcinoma) and MCF-7 cancer cell lines and IC50 values were in the range of 1 to 7 µM. An up-regulation (100-folds) of the early apoptotic markers cfos/cjun, in all the cancer cells, was observed after treatment with AM2.

Other compounds from *Amphidinium* spp. have also been reported earlier to display anticancer activity, such as the cytotoxic macrolides amphinolide G and amphinolide H. These two compounds, especially amphidinolide H, exhibited extremely strong cytotoxic activities against L1210 murine leukemia cells with IC_50_ values of 0.0054 and 0.00048 µg/mL and KB human epidermoid carcinoma cells IC50 values of 0.0059 and 0.00052 µg/mL, respectively [40]. The mechanism of action was related to covalent binding on the actin Tyr200 subdomain [41].

#### 3.3.2. Antifungal Activity

The antifungal activity was assessed by testing amphidinol 22 against *C. albicans* and *A. fumigatus* strains. The compound showed antifungal activity against both fungi, with a MIC value of 64 µg/mL in both cases. Since isolation and identification of the first member of amphidinols’ family [27], almost all the amphidinols discovered to date have presented antifungal activity. Echigoya and co-workers showed a strong antifungal activity on *Aspergillus niger* for AM2, AM4 and AM9 (44.3, 58.2 and 32.9 µg extract per disk, respectively) and a lower activity for AM10, AM11, AM12, AM13 (>100–256.6 µg extract per disk) [20]. In addition, Nuzzo et al. showed that amphidinol 18 (AM18) was active against the fungus *C. albicans* (9 µg/mL) [17]. In 2017, Satake et al. identified the largest amphidinol homologues (AM20 and AM21) [21], but their antifungal activities were low compared to the activities reported for AM6 and AM2 [22].

#### 3.3.3. Antibacterial Activity

The anti-bacterial activity of amphidinol 22 was evaluated as well. Assays were performed on Gram-positive bacteria (*S. aureus* MRSA and *S. aureus* MSSA) and *M. tuberculosis*, and no growth inhibition was observed. Hence, the compound did not display any antibacterial effect.

## 4. Conclusions

Bioassay-guided fractionation allowed the isolation and identification of a new compound belonging to the amphidinol family, which we designated as amphidinol 22, from the dinoflagellate *A. carterae*. It presents different structural features when compared to the other related compounds, including an extra pyran ring and an oxirane group. Bioactivity testing performed for this compound showed that it has cytotoxic and antifungal properties. Amphidinol 22 cytotoxic activity was in range with the already reported activities by Espiritu et al. for AM2 (1 to 7 µM) [29]. On the other hand, AM18 and amphidinol A are the only amphidinols that have been tested for antifungal activity using the MIC assay, and therefore the only studies we can use as a reference. However, the antifungal activity of amphidinol 22 on *C. albicans* (MIC 64 µg/mL) is less potent compared to these molecules (MIC 9 µg/mL for AM18 and 19 µg/mL for amphidinol A) [17,28].

In order to better characterize its mechanism of action, higher amounts of the compound are necessary, as well as further research on biomedical, toxicological, chemical, pharmacological and therapeutic potential. Microalgae have been shown to produce compounds with anticancer and anti-microbial activities and to be cultivable for mass cultivation and massive compound production in photobioreactors [1,12,42,43,44,45,46,47]. Until now, very few pure compounds have been isolated and characterized from microalgae, and they are a still poorly explored resource for drug discovery. This study confirm that they are an excellent reservoir of new marine natural products with applications for various human pathologies. 

## Figures and Tables

**Table 1:** NMR data of amphidinol 22 (500 MHz) in CD3OD.

Carbon	δ^13^C	δ^1^H, mult, *J* (Hz)	Carbon	δ^13^C	δ^1^H, mult, *J* (Hz)
1	14.09	0.91, t, 7.4	43	41.90	1.97, m; 1.51, m
2	23.58	1.42, m, 2H	44	71.79	3.85, m
3	35.83	2.06, m	45	36.68	1.67, m; 1.58, m
4	136.10	5.69, m	46	36.68	2.20, m; 2.11, m
5	131.23	6.05, dd, 15.2, 10.5	47	139.30	Null
6	132.41	6.22, dd, 15.7, 10.4	48	126.24	5.48, br d, 8.7
7	134.08	5.61, dd, 15.2, 8.6	49	67.97	4.56, dd, 8.9, 1.7
8	71.39	4.26, ddd, 6.6, 6.6, 6.6	50	72.50	3.68, dd, 9.5, 1.9
9	40.90	1.75, m, 2H	51	79.21	3.95, m
11	62.32	2.72, dd, 5.2, 2.1	53	67.48	3.97, m
12	71.91	3.41, m	54	30.41	1.76, m
13	35.35	1.62, m; 1.48, m	55	75.71	3.48, m
14	22.89	1.77, m; 1.44, m	56	74.63	3.60, m
15	32.90	1.89, m; 1.44, m	57	32.44	1.96, m; 1.55, m
16	81.54	3.07, m	58	27.89	2.41, m; 2.09, m
17	73.10	3.38, m	59	151.35	Null
18	76.77	3.39, m	60	76.77	4.18, d, 8.9
19	69.89	4.08, m	61	75.23	3.35, m
20	82.36	3.14, br d, 8.9	62	70.41	4.04, m
21	68.63	3.87, m	63	31.55	2.08, m; 1.55, m
22	35.14	2.05, m; 1.74, m	64	67.36	4.05, m
23	77.92	4.22, m	65	68.73	4.04, m
24	71.63	3.64, m	66	80.58	3.74, br d, 9.9
25	67.32	3.92, m	67	72.01	3.97, m
26	30.41	1.76, m	68	74.22	4.37, dd, 7.6, 2.9
27	74.63	3.54, m	69	129.15	5.63, dd, 16.5, 8.0
28	72.59	3.71, m	70	134.97	5.80, m
29	36.76	1.70, m; 1.38, m	71	33.59	2.19, m
30	33.59	1.97, m	72	33.65	2.21, m
31	79.41	3.12, dd, 7.6, 2.8	73	135.94	5.78, m
32	70.50	3.85, m	74	131.97	6.10, dd, 15.2, 10.4
33	46.06	2.29, m	75	134.75	6.21, dd, 15.7, 10.2
34	136.85	null	76	132.68	6.13, dd, 15.7, 10.2
35	129.54	5.72, br s	77	138.61	6.35, ddd, 16.9, 10.2, 10.2
36	144.65	null	78	116.68	5.15, dd, 17.0, 1.0; 5.01, dd, 10.2, 1.0
37	47.72	2.27, m; 2.21, m	79	17.25	0.97, d, 6.8
38	68.68	3.80, m	80	18.51	1.84, br s
39	42.50	1.63, m; 1.27 m	81	115.77	5.05, br s; 4.85, br s
40	31.15	2.12, m	82	13.91	0.90, d, 7.4
41	77.13	3.33, m	83	17.39	1.74, br s
42	72.68	3.64, m	84	113.21	5.07, br s; 4.98 br s

**Table 2:** Percentage of growth inhibition of raw *A. carterae* extracts on cancer cells, bacteria and fungi.

**Anticancer screening ^a^**					
**Cancer cell line**	A549	A2058	HepG2	MCF7	MiaPaca-2
**% Growth Inhibition**	100	100	100	100	100
**Antibacterial screening ^b^**					
**Bacteria**	*E. coli*	*K. pneumoniae*	MRSA	MSSA	*M. tuberculosis*
**% Growth Inhibition**	37	19	95	88	83
**Antifungal screening ^b^**					
**Fungus**	*A. fumigatus*				
**% Growth Inhibition**	100				

^a^ Tested at a concentration of 175 µg/mL. ^b^ Tested at a concentration of 560 µg/mL. The values are the mean of three biological replicates and two technical replicates.

## Supplementary Materials

The following are available online at https://www.mdpi.com/1660-3397/17/7/385/s1, Figure S1: 1H NMR spectrum of amphidinol 22 (500 MHz) in CD3OD, Figure S2: 13C NMR spectrum of amphidinol 22 (125 MHz) in CD3OD, Figure S3: HSQC spectrum of amphidinol 22, Figure S4: COSY spectrum of amphidinol 22, Figure S5: HMBC spectrum of amphidinol 22, Figure S6: NOESY spectrum of amphidinol 22, Figure S7: LC-UV trace and UV and HRESIMS spectra of amphidinol 22, Figure S8: Expansions of the HRESIMS spectrum of amphidinol 22, and Table S9: Tabulated 2D NMR data of amphidinol 22.
