# Supplementary material for: Amphidinol 22, a New Cytotoxic and Antifungal Amphidinol from the Dinoflagellate Amphidinium carterae
Source: Mar Drugs. 2019 Jun 27;17(7):385. doi: 10.3390/md17070385 (PMC6669446; doi:10.3390/md17070385)
Supplement: Supplementary file 1 [file marinedrugs-17-00385-s001.pdf]

Supplementary information

Figure S1. ^{1}H NMR spectrum of amphidinol 22 (500 MHz) in CD_3OD. 2

Figure S2. ^{13}C NMR spectrum of amphidinol 22 (125 MHz) in CD_3OD. 3

Figure S3. HSQC spectrum of amphidinol 22. 4

Figure S4. COSY spectrum of amphidinol 22. 5

Figure S5. HMBC spectrum of amphidinol 22... 6

Figure S6. NOESY spectrum of amphidinol 22. 7

Figure S7. LC-UV trace and UV and HRESIMS spectra of amphidinol 22........................ 8

Figure S8. Expansions of the HRESIMS spectrum of amphidinol 22................................. 9

Table S9. Tabulated 2D NMR data of amphidinol 22 10

Figure S1. ^{1}H NMR spectrum of amphidinol 22 (500 MHz) in CD_3OD.

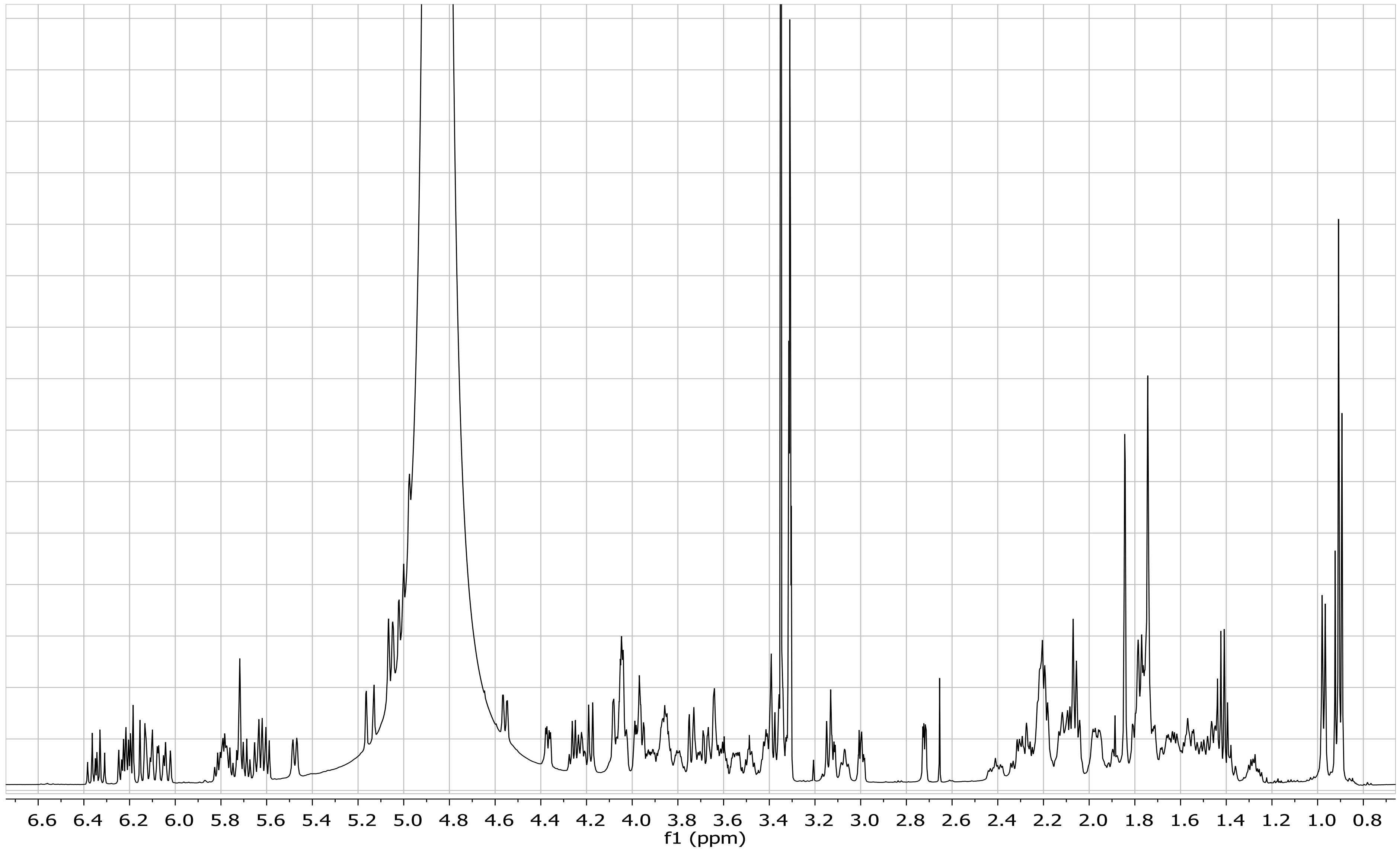

Figure S2. ^{13}C NMR spectrum of amphidinol 22 (125 MHz) in CD_3OD.

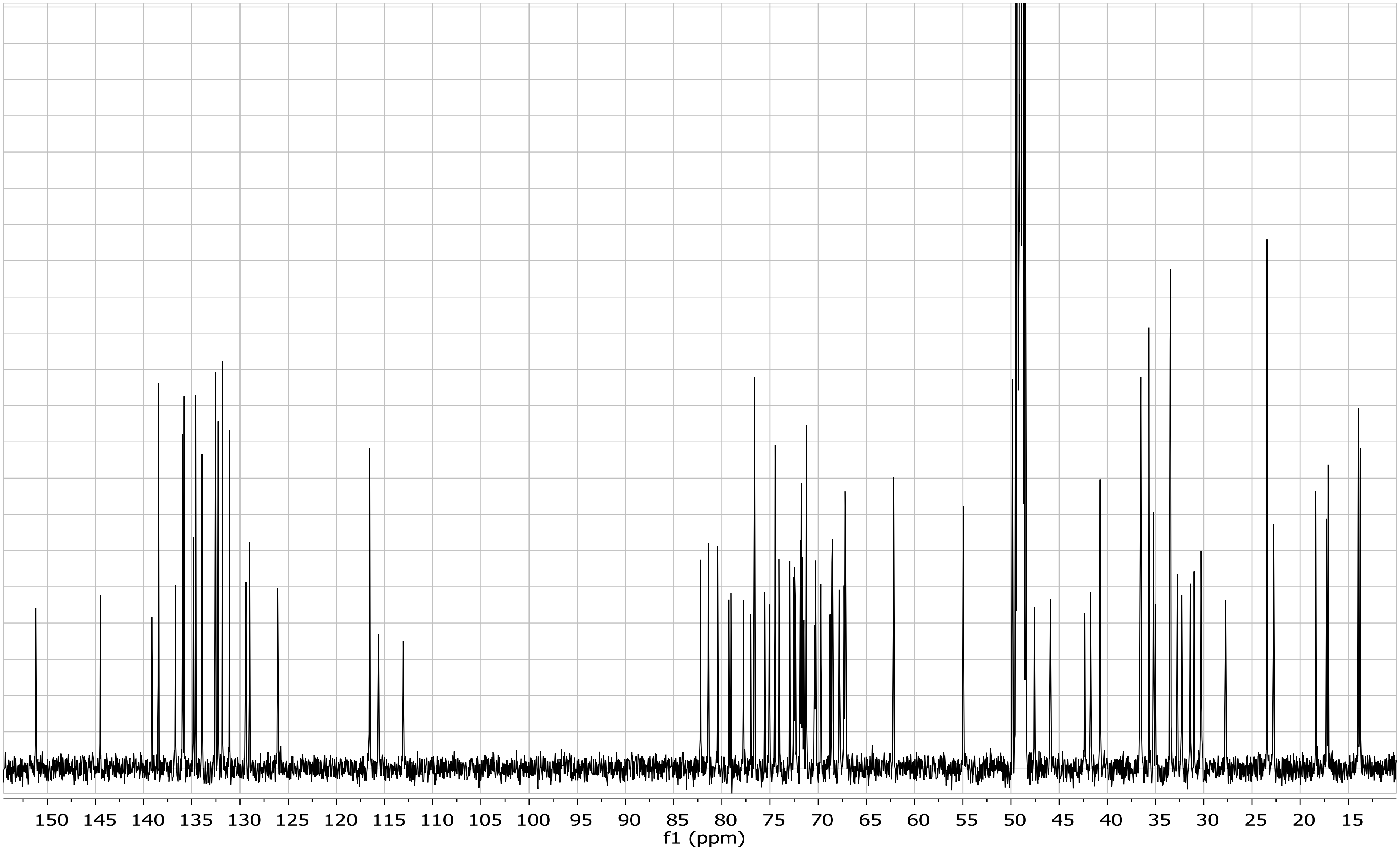

Figure S3. HSQC spectrum of amphidinol 22.

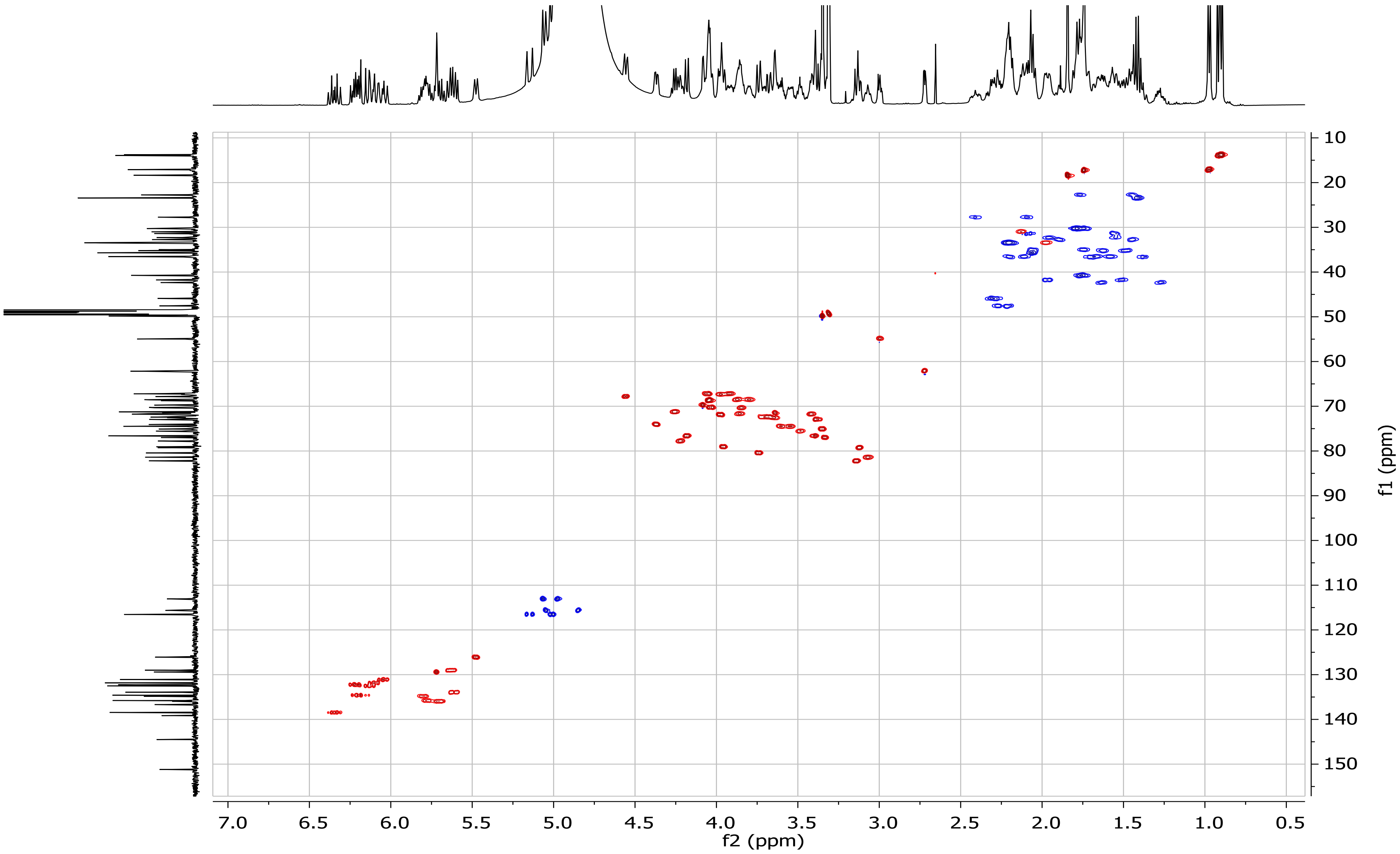

Figure S4. COSY spectrum of amphidinol 22.

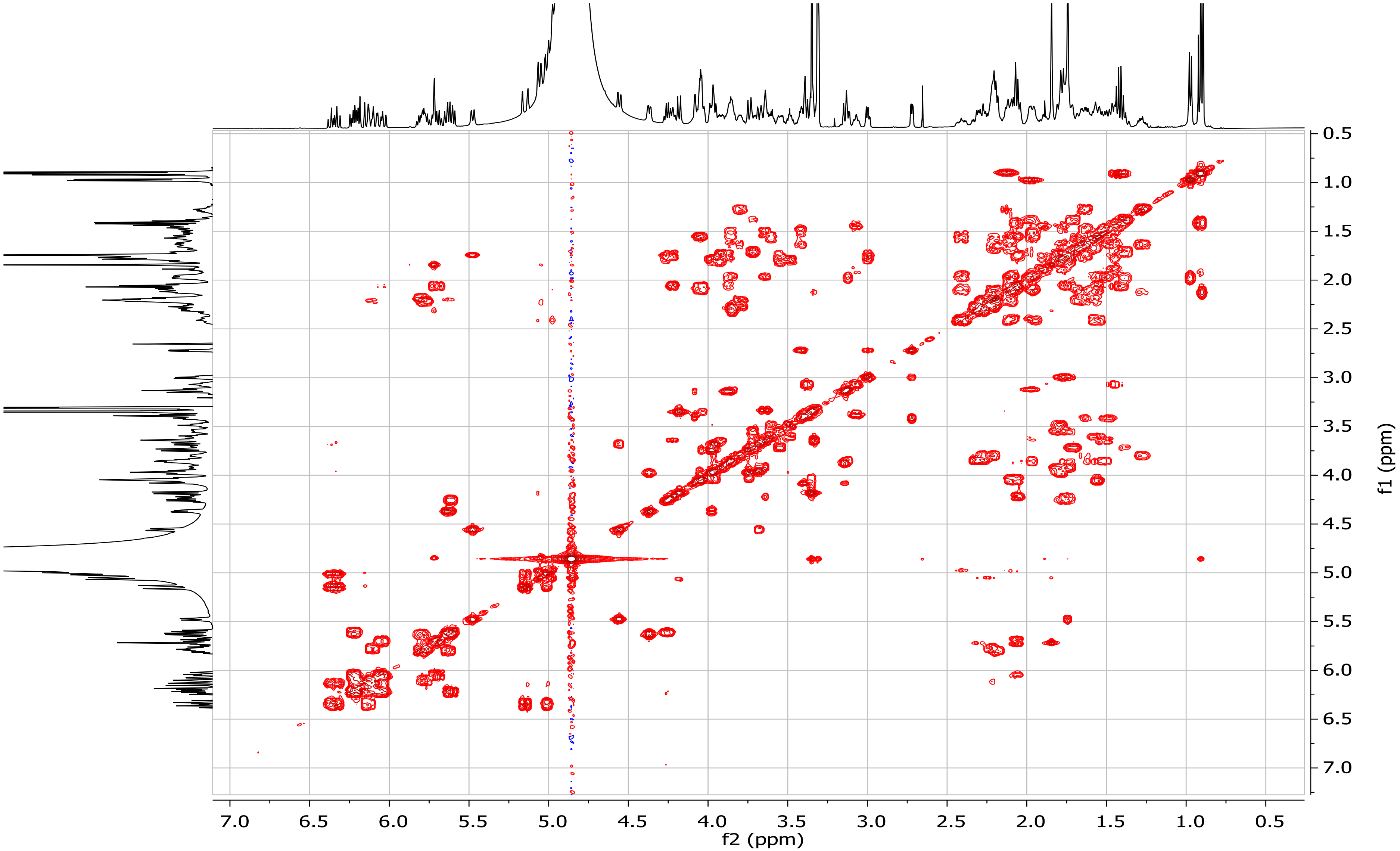

Figure S5. HMBC spectrum of amphidinol 22

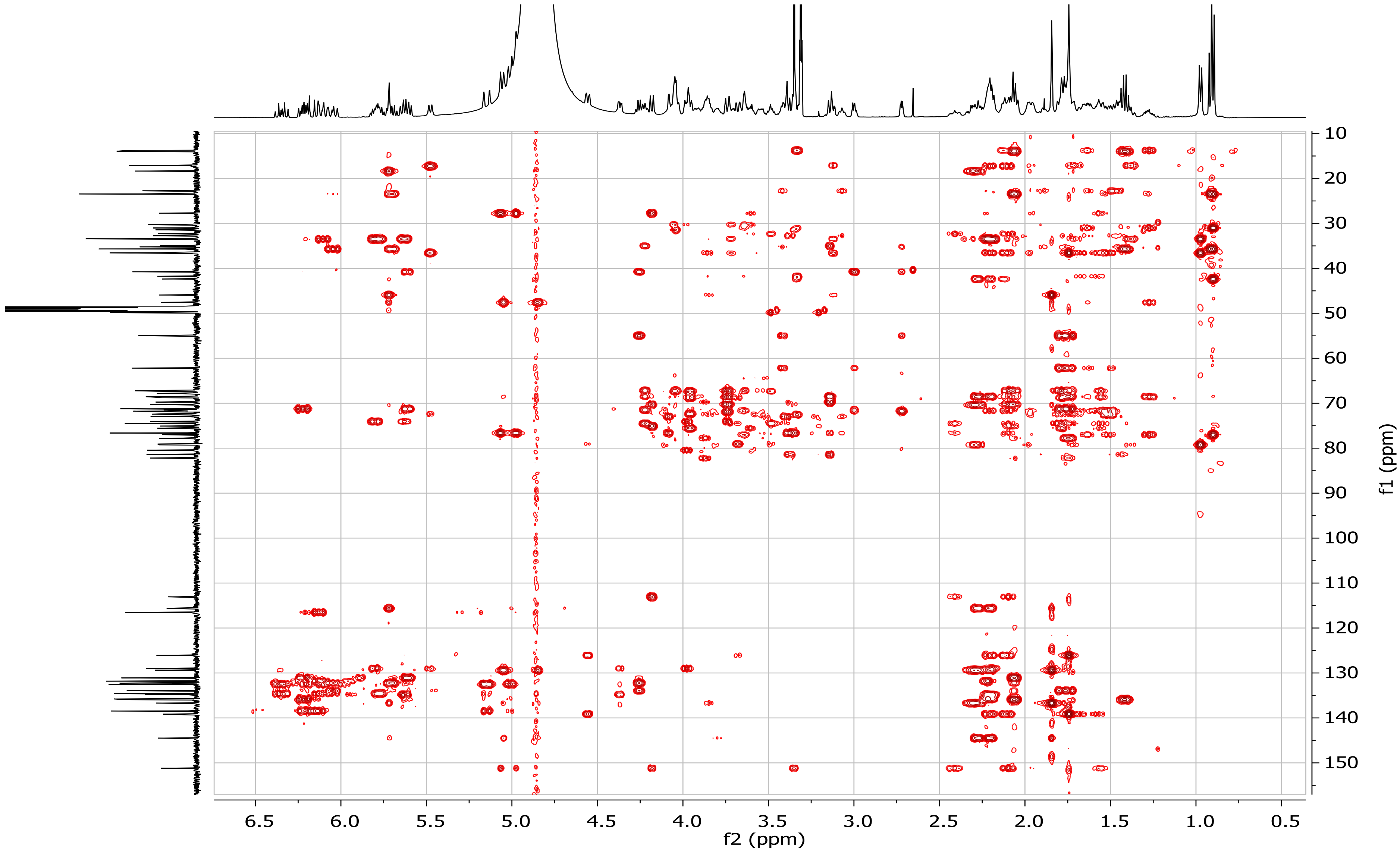

Figure S6. NOESY spectrum of amphidinol 22.

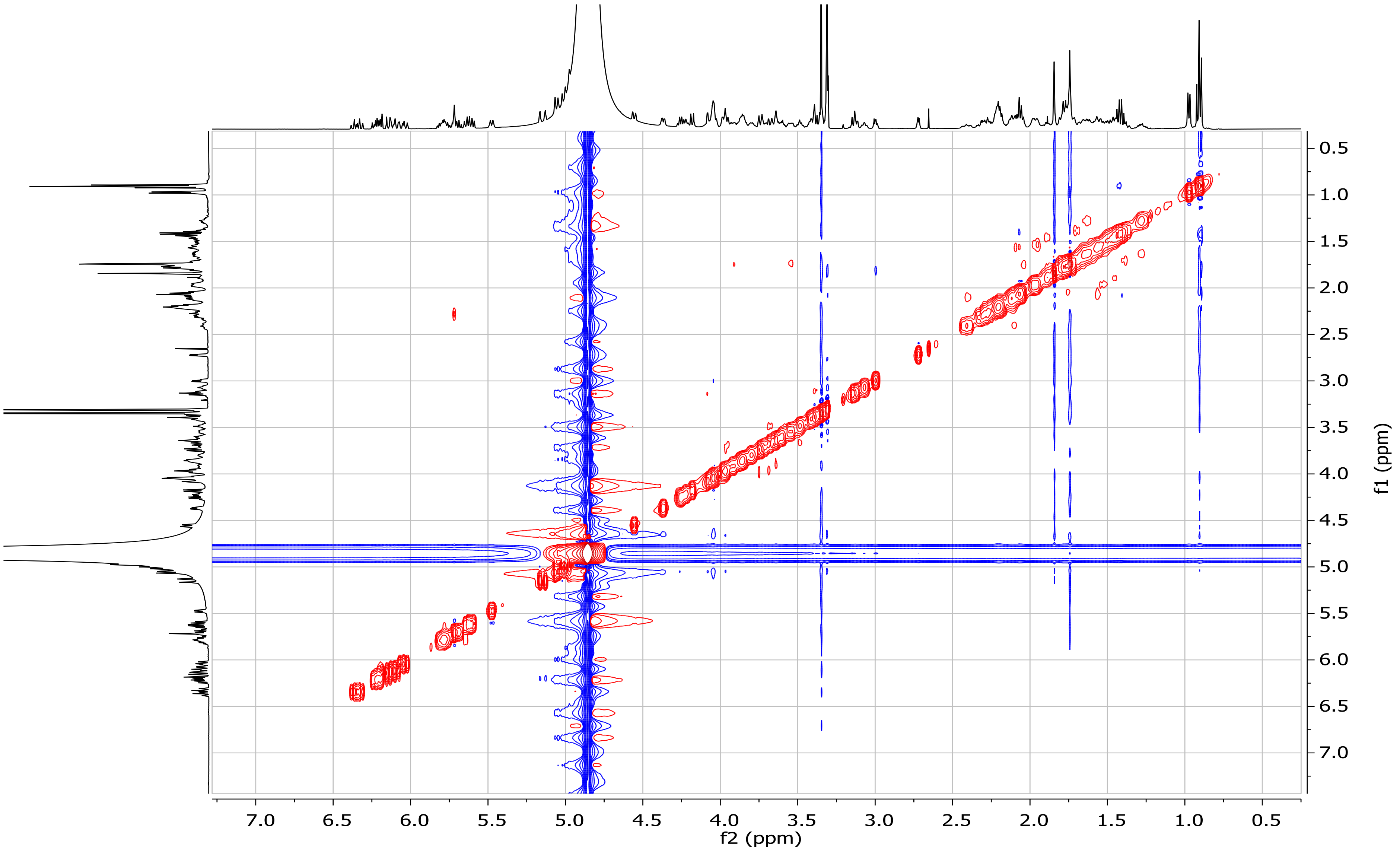

Figure S7. LC-UV trace and UV and HRESIMS spectra of amphidinol 22

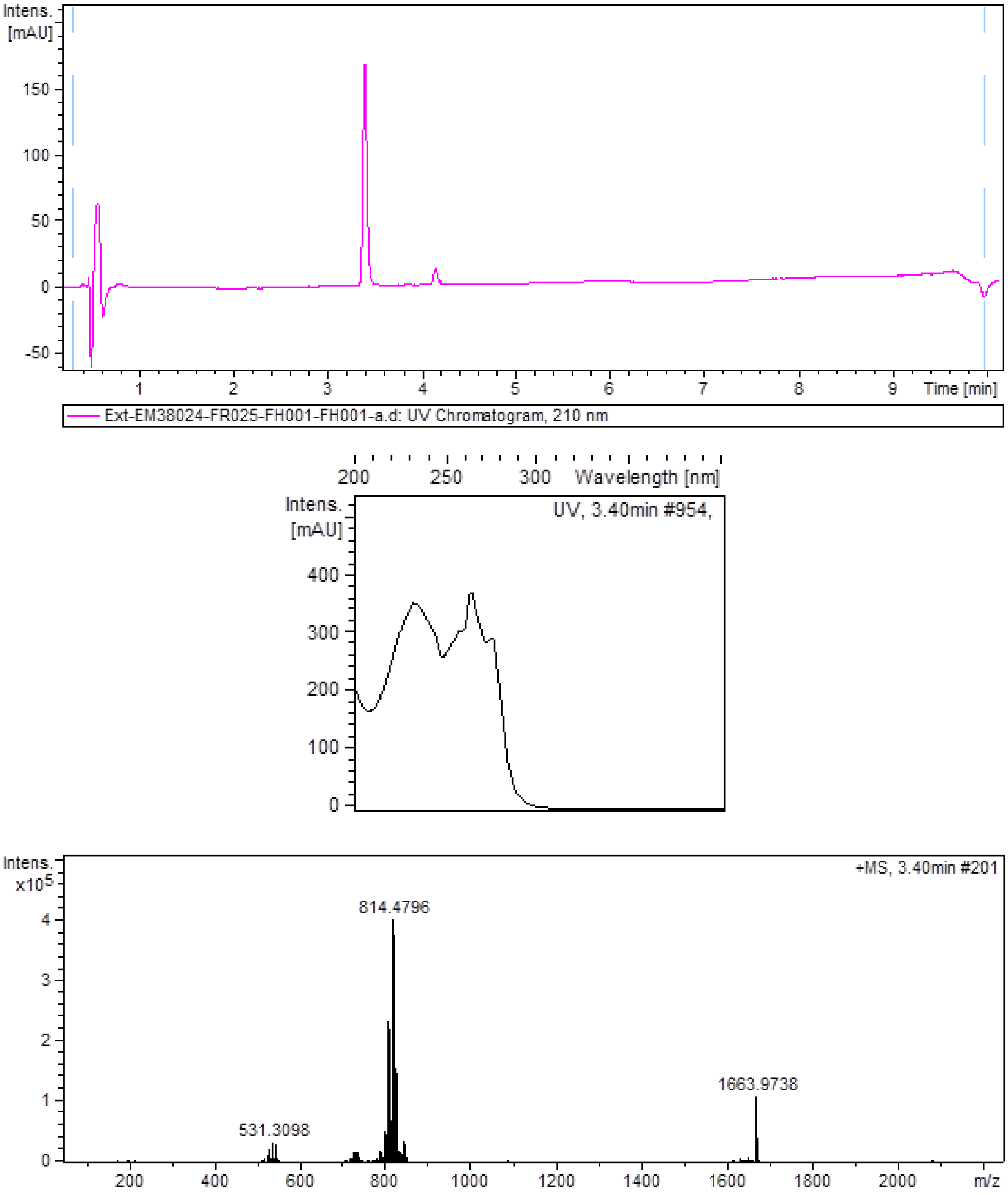

Figure S8. Expansions of the HRESIMS spectrum of amphidinol 22

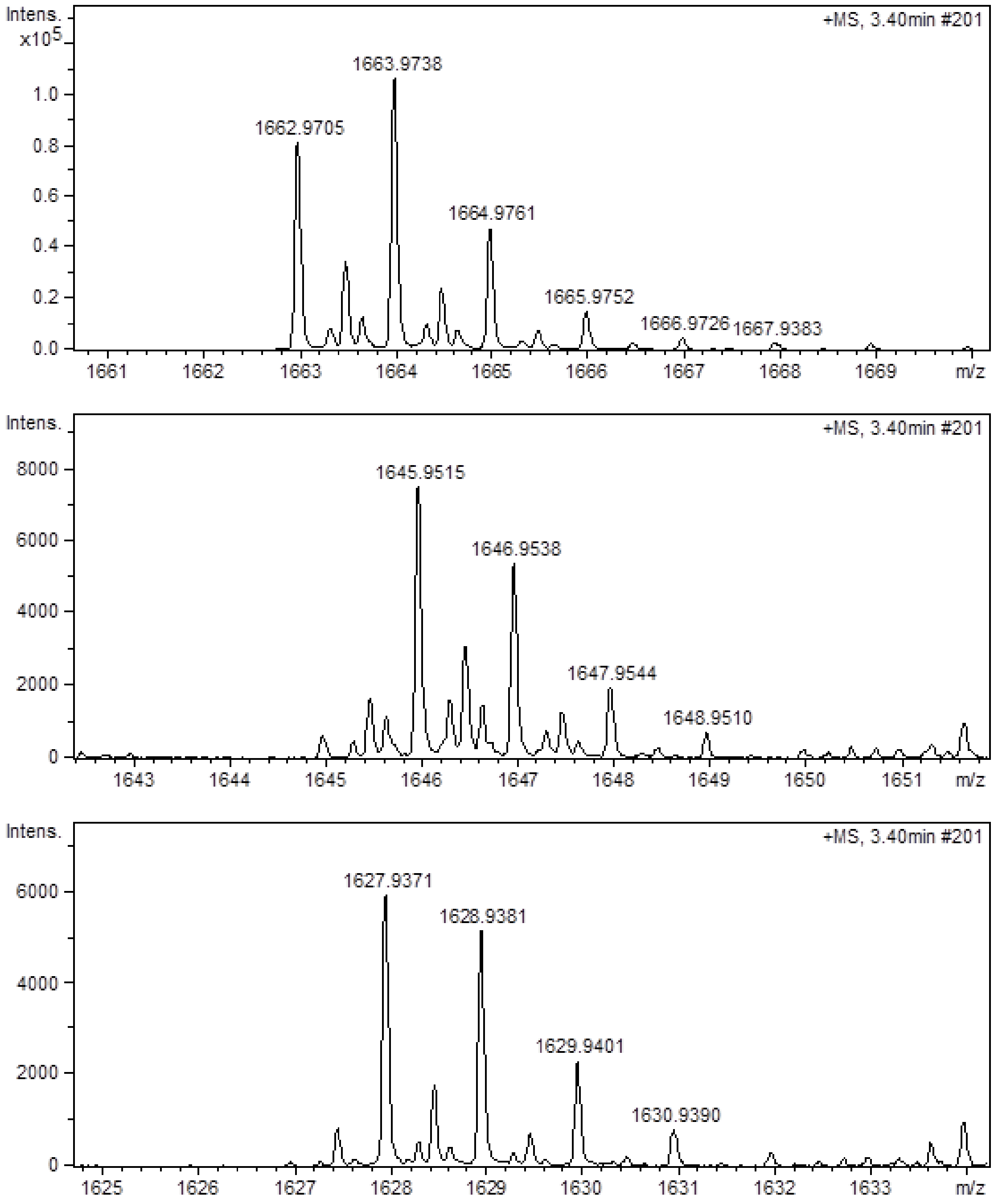

Table S9. Tabulated 2D NMR data of amphidinol 22

Carbon	δ¹H, mult, *J* (Hz)	COSY (δ¹H)	HMBC (δ¹³C)	Carbon	δ¹H, mult, *J* (Hz)	COSY (δ¹H)	HMBC (δ¹³C)
1	0.91, t, 7.4	1.42	23.58, 35.83	43	1.97, m; 1.51, m	3.64, 3.85	71.79, 77.13
2	1.42, m, 2H	0.91, 2.06	14.09, 35.83, 136.10	44	3.85, m	1.97, 1.51, 1.67, 1.58	36.68, 41.90
3	2.06, m	1.42, 5.69, 6.05 (ld)	14.09, 23.58, 136.10, 131.23	45	1.67, m; 1.58, m	2.20, 2.11, 3.86	36.68, 41.90, 71.79, 139.30
4	5.69, m	2.06, 6.05	23.58, 35.83, 131.23, 132.41	46	2.20, m; 2.11, m	1.67, 1.58	17.39, 36.68, 71.79, 126.24, 139.30
5	6.05, dd, 15.2, 10.5	5.69, 6.22	35.83, 134.08, 132.41	47	null	-	-
6	6.22, dd, 15.7, 10.4	5.61, 6.05	131.23, 136.10	48	5.48, br d, 8.7	4.56, 1.74(ld)	17.39, 36.68, 72.50
7	5.61, dd, 15.2, 8.6	6.22, 4.26	40.90, 71.39, 131.23	49	4.56, dd, 8.9, 1.7	5.48, 3.68	79.21, 126.24, 139.30
8	4.26, ddd, 6.6, 6.6, 6.6	1.75, 5.61	40.90, 55.11, 132.1, 134.08	50	3.68, dd, 9.5, 1.9	4.56, 3.95	68.92, 79.21, 126.24
9	1.75, m, 2H	3.00, 4.26	55.11, 62.32, 71.39, 134.08	51	3.95, m	3.68, 4.05	67.48, 68.92, 72.50, 75.71
10	3.00, ddd, 6.2, 5.6, 2.1	2.72, 1.75	40.90, 62.32, 71.91	52	4.05, m	3.95, 3.97	30.41
11	2.72, dd, 5.2, 2.1	3.00, 3.41	35.35, 40.90, 55.11, 71.91	53	3.97, m	4.05, 1.76	-
12	3.41, m	2.72, 1.62, 1.48	22.89, 35.35, 55.11, 62.32	54	1.76, m	3.97, 3.48	67.48, 68.92, 74.63, 75.71
13	1.62, m; 1.48, m	3.41, 1.77, 1.44	22.89, 32.90, 62.32	55	3.48, m	1.76, 3.60	32.44, 67.48, 74.63, 79.21
14	1.77, m; 1.44, m	1.62, 1.48, 1.89, 1.44	35.35	56	3.60, m	3.48, 1.96, 1.55	27.89, 30.41, 32.44, 75.71
15	1.89, m; 1.44, m	1.76, 1.44, 3.07	22.89, 35.35, 73.10, 81.54	57	1.96, m; 1.55, m	3.60, 2.41, 2.09	27.89, 74.63, 75.71, 151.35
16	3.07, m	1.89, 1.44, 3.38	22.89, 32.90, 73.10, 76.77	58	2.41, m; 2.09, m	1.96, 1.55	32.44, 74.63, 76.77, 113.21, 151.35
17	3.38, m	3.07, 3.39	32.90, 81.54, 69.89	59	null	-	-

18	3.39, m	3.38, 4.08	73.10	60	4.18, d, 8.9	3.35	27.89, 70.41, 75.23, 113.21, 151.35
19	4.08, m	3.39, 3.14	73.10, 76.77	61	3.35, m	4.18, 4.04	31.55, 76.77, 151.35
20	3.14, br d, 8.9	4.08, 3.87	35.14, 68.63, 69.89, 76.77, 81.54	62	4.04, m	3.35, 2.08, 1.55, 3.74	31.55, 67.36
21	3.87, m	3.14, 2.05, 1.74	35.14, 69.89, 77.92, 82.36	63	2.08, m; 1.55, m	4.00 to 4.09 (broad signals)	67.36, 68.73, 70.41
22	2.05, m; 1.74, m	3.87, 4.22	68.63, 77.92, 82.36	64	4.05, m	3.35, 2.08, 1.55, 3.74	31.55
23	4.22, m	2.05, 1.74, 3.64	35.14, 67.32, 68.63, 71.63, 74.63	65	4.04, m	3.35, 2.08, 1.55, 3.74	67.36, 31.55
24	3.64, m	4.22, 3.92	30.41, 67.32	66	3.74, br d, 9.9	4.04, 3.97	67.36, 68.73, 70.41, 72.02, 74.22
25	3.92, m	3.64, 1.76	30.41, 71.63	67	3.97, m	3.74, 4.37	74.22, 80.58, 129.15
26	1.76, m	3.92, 3.54	67.32, 74.63	68	4.37, dd, 7.6, 2.9	3.97, 5.63	129.15, 134.97
27	3.54, m	1.76, 3.72	36.76, 67.32, 72.59	69	5.63, dd, 16.5, 8.0	4.37, 5.80	33.59, 74.22, 134.97
28	3.71, m	3.54, 1.70, 1.38	30.41, 33.59, 36.76	70	5.80, m	5.63, 2.19	33.59, 33.65, 74.22, 129.15
29	1.70, m; 1.38, m	3.71, 1.97	17.25, 33.59, 74.63, 79.41	71	2.19, m	5.80	33.65, 129.15, 134.97
30	1.97, m	1.70, 1.38, 3.12, 0.97	17.25, 36.76, 72.59, 79.41	72	2.21, m	5.78	33.59, 131.97, 135.94
31	3.12, dd, 7.6, 2.8	1.97, 3.85	17.25, 33.59, 36.76, 46.06	73	5.78, m	2.21, 6.10	33.59, 33.65, 134.75
32	3.85, m	3.12, 2.29	46.06, 136.85	74	6.10, dd, 15.2, 10.4	5.78, 6.25	33.65, 132.68
33	2.29, m	3.85	18.51, 70.50, 79.41, 129.54, 136.85	75	6.21, dd, 15.7, 10.2	6.10, 6.13	132.68, 138.61
34	null	-	-	76	6.13, dd, 15.7, 10.2	6.21, 6.35	116.68, 134.75, 138.61
35	5.72, br s	1.84(ld), 4.85(ld)	18.51, 46.06, 47.72, 115.77, 136.85	77	6.35, ddd, 16.9, 10.2, 10.2	6.13, 5.15, 5.01	132.68, 134.75
36	null	-	-	78	5.15, dd, 17.0, 1.0; 5.01, dd, 10.2, 1.0	6.35	132.68, 134.75, 138.61

37	2.27, m; 2.21, m	3.80, 5.05(ld)	42.50, 68.68, 115.77, 129.54	79	0.97, d, 6.8	1.97	33.59, 36.76, 79.41
38	3.80, m	2.27, 2.21, 1.63, 1.27	31.15, 47.72, 144.65	80	1.84, br s	2.29(ld), 5.72(ld)	46.06, 47.72, 115.77, 129.54, 136.85, 144.65
39	1.63, m; 1.27, m	3.80, 2.12	13.91, 31.15, 47.72, 68.68, 77.13	81	5.05, br s; 4.85, br s	2.27(ld), 2.21(ld), 1.84(ld)	47.72, 129.54, 144.65
40	2.12, m	1.63, 1.27, 3.33, 0.90	13.91, 42.50, 77.13	82	0.90, d, 7.4	2.12	31.15, 42.50, 77.13
41	3.33, m	2.12, 3.64	13.91, 31.15, 42.50, 72.68	83	1.74, br s	5.47(ld)	36.68, 126.24, 139.30
42	3.64, m	1.97, 1.51, 3.33	41.90, 71.79, 77.13	84	5.07, br s; 4.98, br s	-	27.89, 76.77, 151.35

* Signals marked in red are not belonging to the protons where they were assigned, but it was really difficult to distinguish them only with the COSY data.
